# Multiple Low Energy Long Bone Fractures in the Setting of Rothmund-Thomson Syndrome

**DOI:** 10.1155/2015/495164

**Published:** 2015-11-05

**Authors:** Nicholas Beckmann

**Affiliations:** University of Texas Health Science Center, 6431 Fannin Street, MSB 2.130B, Houston, TX 77030, USA

## Abstract

Rothmund-Thomson syndrome is a rare autosomal recessive genodermatosis characterized by a poikilodermatous rash starting in infancy as well as various skeletal anomalies, juvenile cataracts, and predisposition to certain cancers. Although Rothmund-Thomson syndrome is associated with diminished bone mineral density in addition to multiple skeletal abnormalities, there are few reports of the association with stress fractures or pathologic fractures in low energy trauma or delayed healing of fractures. Presented is a case of a young adult male with Rothmund-Thomson syndrome presenting with multiple episodes of long bone fractures caused by low energy trauma with one of the fractures exhibiting significantly delayed healing. The patient was also found to have an asymptomatic stress fracture of the lower extremity, another finding of Rothmund-Thomson syndrome rarely reported in the literature. A thorough review of the literature and comprehensive presentation of Rothmund-Thomson syndrome is provided in conjunction with our case.

## 1. Case Report

An 18-year-old male with history of Rothmund-Thomson syndrome diagnosed at the age of two presented with acute right mid leg pain while cutting to kick the ball during a soccer game. He reported a history of prior right leg fracture four years ago sustained while playfully wrestling with his younger brother. After the prior fracture healed, the patient noticed a bump at the level of his mid right shin, which corresponded to the site of the patient's current leg pain. He denied any pain in the right leg prior to sustaining the fracture, and he denied any other preexisting medical conditions.

**Figure 1:**
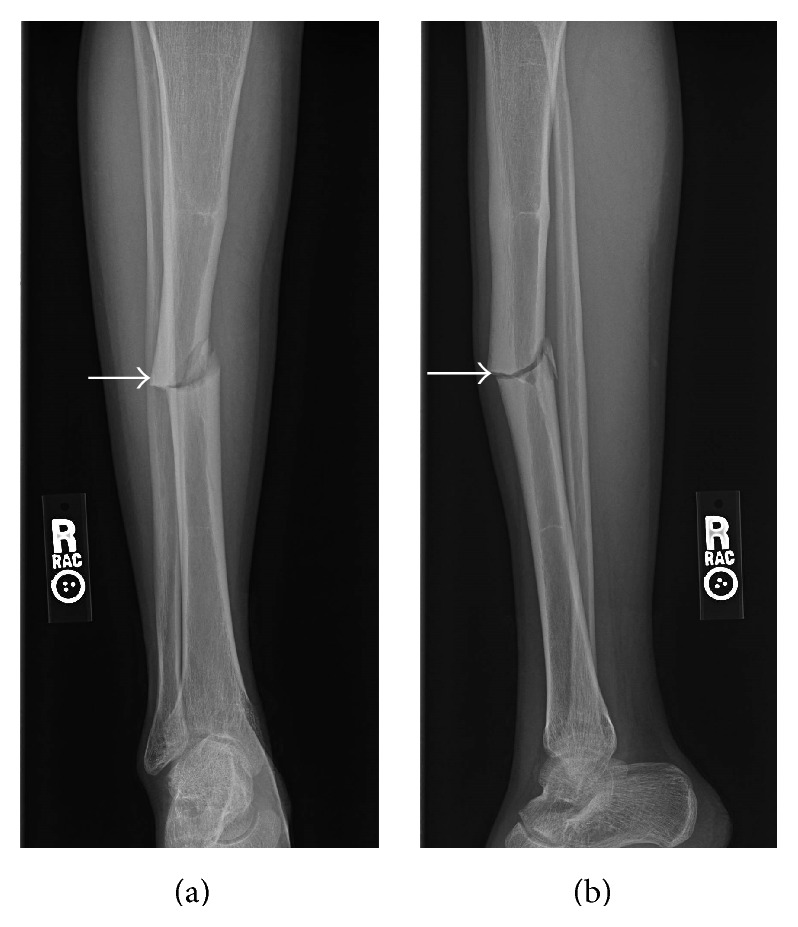
Young adult male with history of Rothmund-Thomson syndrome diagnosed at 2 years of age. Findings: (a) mildly comminuted and mildly displaced fracture through the mid diaphysis of the right tibia. There is lateral cortical thickening at the level of the fracture (arrow). (b) Mildly displaced fracture through the mid tibia diaphysis with focal anterior cortical thickening at the level of the fracture (arrow). Technique: (a) AP radiograph kVp = 66, mAs = 2, and (b) lateral radiograph kVp = 64, mAs = 2.

**Figure 2:**
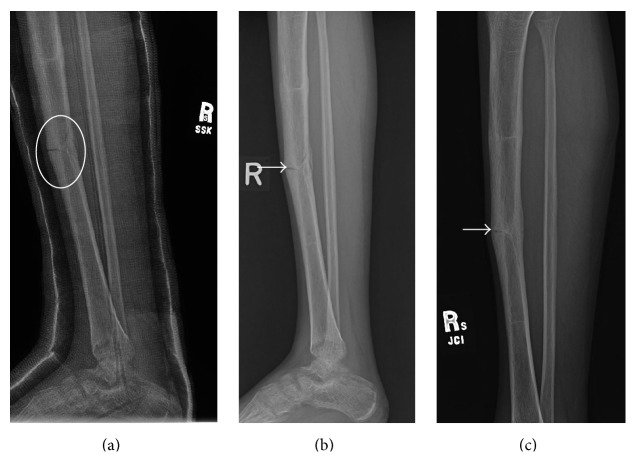
Young adult male with history of Rothmund-Thomson syndrome diagnosed at 2 years of age. Findings: (a) follow-up radiograph 5 months after injury shows delayed union of the tibia diaphyseal fracture with less than 50% bridging bone at the fracture (oval). (b) 7-month follow-up radiograph shows approximately 50% bridging bone at the tibia diaphyseal fracture with fracture line still easily visualized anteriorly (arrow). (c) Final follow-up radiograph obtained 13 months after initial injury shows near-complete bridging bone across the fracture with fracture line still faintly apparent anteriorly (arrow). Technique: (a) lateral radiograph kVp = 60, mAs = 4, (b) lateral radiograph kVp = 63, mAs = 3, and (c) lateral radiograph kVp = 60, mAs = 3.

**Figure 3:**
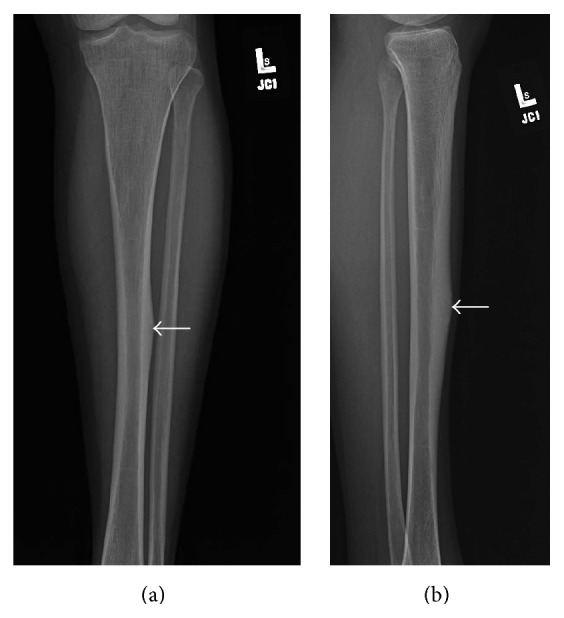
Young adult male with history of Rothmund-Thomson syndrome diagnosed at 2 years of age. Findings: (a) focal thickening of the lateral cortex of the mid left tibia diaphysis (arrow) is visible on AP radiograph obtained at the same time as the 13-month follow-up images of the right tibia fracture. No fracture or underlying lytic bone lesion is apparent. (b) Lateral radiograph of the left tibia shows anterior cortical thickening (arrow). Technique: (a) AP radiograph kVp = 63, mAs = 3, and (b) lateral radiograph kVp = 63, mAs = 3.

Presenting radiographs of the right tibia-fibula showed mild deformity of the right tibia from prior healed fracture with mildly displaced and comminuted fracture through the mid diaphysis of the tibia (Figure 1). Focal anterior cortical thickening of the tibia was present at the level of the acute fracture. The fracture was treated with closed reduction and casting. Serial follow-up radiographs obtained over the next 13 months showed delayed and incomplete union across the tibia fracture (Figure 2). At the 13-month follow-up, additional radiographs of the left tibia were obtained to evaluate a focal painless bump of the mid left shin the patient reported developing shortly after his right tibia fracture. Left tibia radiographs showed focal anterior cortical thickening of the mid tibia diaphysis corresponding to the palpable bump (Figure 3). No fracture line or lytic bone lesion was appreciated. The patient did not return for additional follow-up.

**Figure 4:**
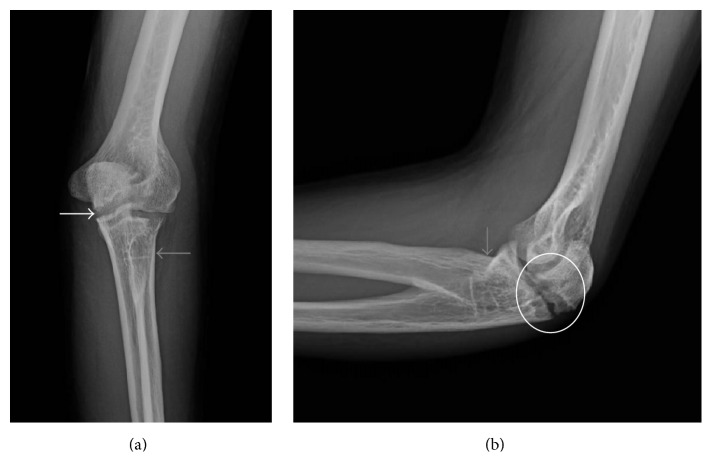
Young adult male with history of Rothmund-Thomson syndrome diagnosed at 2 years of age. Findings: (a) mildly displaced fracture through the olecranon (white arrow). There is congenital fusion of radial head and proximal ulna (grey arrow). (b) The mildly displaced olecranon is better appreciated on the lateral radiograph. There is osteolysis and cortication along the fracture margins (oval), which raised the concern for underlying lytic bone lesion. Congenital fusion of the radial head and ulna is again noted (grey arrow). Technique: (a) AP radiograph kVp = 65, mAs = 4, and (b) Lateral radiograph kVp = 65, mAs = 4.

**Figure 5:**
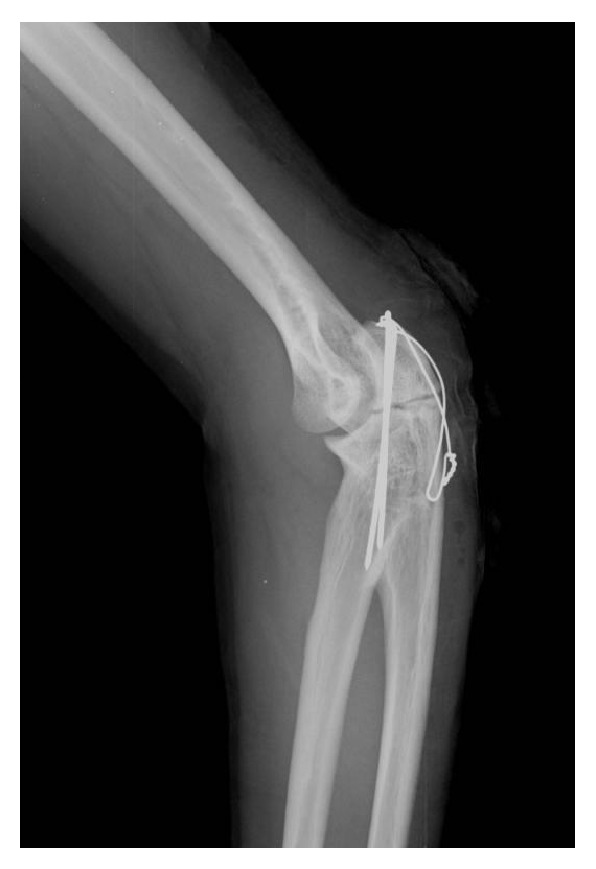
Young adult male with history of Rothmund-Thomson syndrome diagnosed at 2 years of age. Findings: there is satisfactory alignment of the olecranon fracture after tension band wiring. Technique: lateral radiograph kVp = 60, mAs = 3.

**Figure 6:**
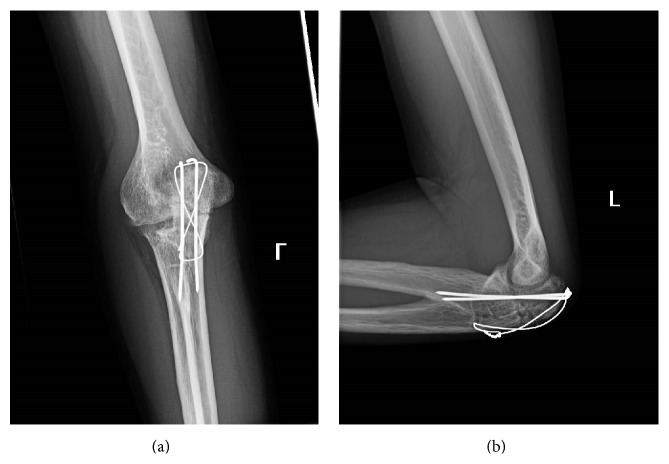
Young adult male with history of Rothmund-Thomson syndrome diagnosed at 2 years of age. Findings: no bridging bone formation had occurred at the two-month follow-up radiograph of the olecranon fracture. Technique: lateral radiograph kVp = 60, mAs = 2.

The patient returned to clinic 4 years later for treatment of a left olecranon fracture sustained during a fall playing soccer 3.5 weeks earlier. Presenting radiographs showed a mildly displaced subacute fracture through the left olecranon (Figure 4). There was early cortication along the fracture margins and more osteolysis than expected along the fracture line given the reported age of the fracture. Therefore, a bone biopsy of the fracture was performed, which was negative for neoplasm and infection. Tension band wiring of the olecranon fracture was subsequently performed (Figure 5). Early remodeling without bridging bone formation was present on two-month follow-up radiographs (Figure 6). The patient did not return for any additional follow-up of his elbow fracture.

**Figure 7:**
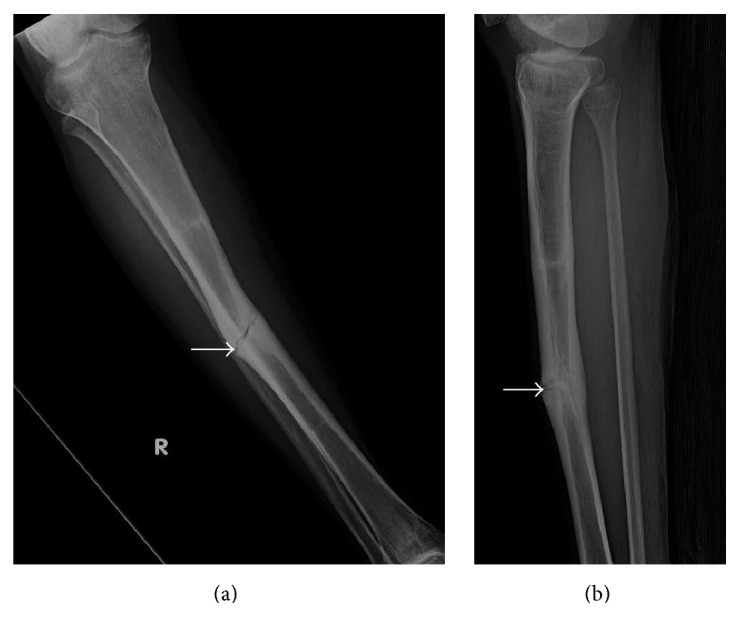
Young adult male with history of Rothmund-Thomson syndrome diagnosed at 2 years of age. Findings: (a) recurrent mildly displaced mid tibia diaphyseal fracture through the area of tibia fracture 9 years earlier (arrow). (b) Mildly displaced fracture of the mid tibia diaphysis again seen. There is increased cortical thickening of the mid tibia diaphysis (arrow). Technique: (a) AP radiograph kVp = 57, mAs = 3, and (b) lateral radiograph kVp = 57, mAs = 3.

**Figure 8:**
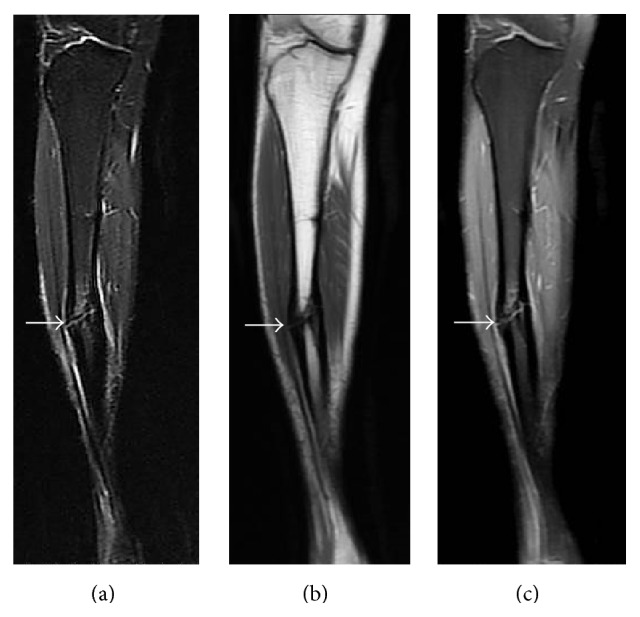
Young adult male with history of Rothmund-Thomson syndrome diagnosed at 2 years of age. Findings: (a) recurrent mildly displaced right tibia diaphyseal fracture (arrow) with mild associated bone marrow edema and adjacent soft tissue edema. (b) Cortical thickening is present at the fracture site (arrow). There is no mass-like bone lesion identified, and normal fatty marrow signal extends almost to the fracture line. (c) Mild bone marrow and periosteal enhancement is present at the fracture site (arrow). No nodular or mass-like enhancement is present. Technique: (a) coronal STIR MRI (TR = 6350, TE = 44.5), ST = 5 mm, spacing = 6 mm, FOV = 38.0 × 44.8 cm, and matrix = 224 × 320; (b) coronal T1 MRI (TR = 650, TE = 13.2), ST = 5 mm, spacing= 6 mm, FOV = 38.0 × 44.8 cm, and matrix = 224 × 352; (c) coronal T1 postcontrast fat-saturated MRI (TR = 650, TE = 12.3), ST = 5 mm, spacing = 6 mm, FOV = 38.0 × 44.8 cm, matrix 224 × 320, and contrast = 13 mL, Magnevist.

The patient again returned to clinic approximately 5 years following the olecranon fracture with acute right mid leg pain after landing on the right leg awkwardly while jumping during a soccer game. Radiographs showed a mildly displaced acute fracture through the area of prior fracture 9 years earlier (Figure 7). An MRI with and without contrast of the right leg was performed to exclude underlying malignancy at the fracture (Figure 8). No bone tumor was present on the MRI.

**Figure 9:**
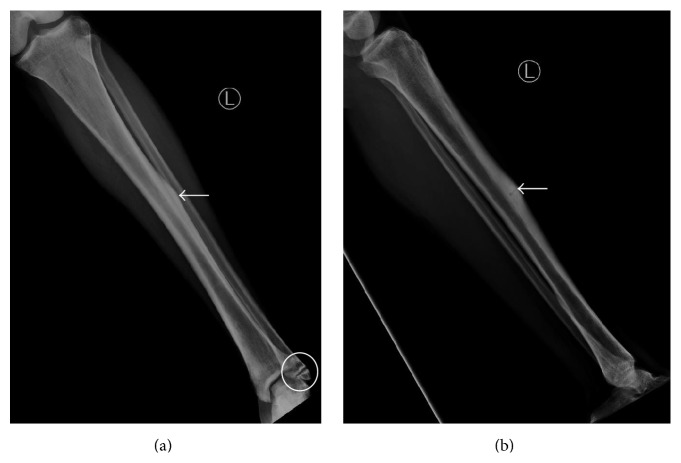
Young adult male with history of Rothmund-Thomson syndrome diagnosed at 2 years of age. Findings: (a) increased cortical thickening of the lateral mid tibia diaphysis when compared to prior radiographs from 8 years earlier (arrow). A chronic appearing ununited lateral malleolus fracture is also present (circle). (b) An increase in anterior cortical thickening of the mid tibia diaphysis has also occurred with a subtle fracture line through the anterior tibial cortex at the site of cortical thickening (arrow). Technique: (a) AP radiograph kVp = 63, mAs = 3, and (b) lateral radiograph kVp = 63, mAs = 3.

Repeat radiographs of the left tibia-fibula were also obtained due to the patient reporting a gradual increase in size of the bump on his left shin he had first noticed eight years earlier. The radiographs showed increase in the focal cortical thickening at the anterior mid tibia with development of a stress fracture through the area of cortical thickening (Figure 9). An old ununited transverse fracture through the left lateral malleolus was incidentally noted on the tibia-fibula images (Figure 9). Upon interviewing the patient again, he reported injuring his left ankle 6 years earlier while stepping off a truck. He treated the ankle injury with compression dressing without seeking professional medical treatment. He could not recall any other trauma to the left ankle.

**Figure 10:**
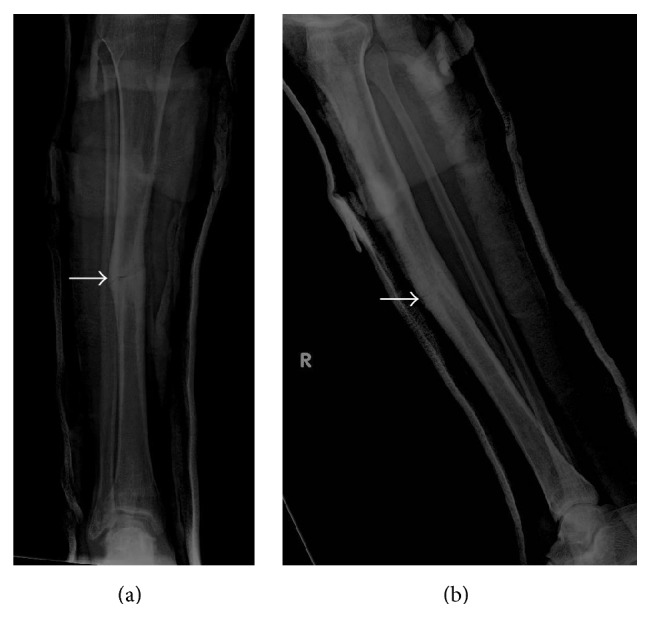
Young adult male with history of Rothmund-Thomson syndrome diagnosed at 2 years of age. Findings: (a) satisfactory alignment of the now minimally displaced mid tibia diaphyseal fracture (arrow) on the AP projection after casting. (b) Lateral view also shows satisfactory alignment of the tibia diaphyseal fracture (arrow) after casting. Technique: (a) AP radiograph kVp = 60, mAs = 3, and (b) lateral radiograph kVp = 60, mAs = 4.

The patient was treated with closed reduction and casting of the right tibia fracture (Figure 10), and he was discharged on crutches to remain on non-weight bearing on the right leg for 6 weeks. Since the patient's left tibia stress fracture was asymptomatic, no treatment recommendations for the stress fracture were made.

## 2. Discussion

### 2.1. Introduction

Rothmund-Thomson syndrome (RTS) was first described in 1868 by Auguste Rothmund in inbred family members with an unusual rash and juvenile cataracts [1]. Sydney Thomson in 1923 described a condition in patients with a similar rash and skeletal anomalies but no cataracts, which he called “poikiloderma congenitale” [2]. In 1957, Taylor postulated that these two syndromes share the same pathophysiology and proposed the name Rothmund-Thomson syndrome [3]. RTS is a rare genetic disorder with only a few hundred cases reported in the literature. RTS typically presents with a poikilodermatous rash in infancy in conjunction with a wide array of other dermal, skeletal, and gastrointestinal findings. While RTS is associated with diminished bone mineral density creating a potential risk for pathologic and stress related fractures, there is little literature discussing the occurrence of fractures in RTS patients. This case illustrates the risk of stress and acute insufficiency fractures in a patient with diffusely demineralized bones related to RTS.

### 2.2. Etiology and Incidence

RTS is a rare autosomal recessive disorder. It is frequently, but not always, associated with a mutation in the* RECQL4* gene. This association was first discovered in 1999 [4] and has led to RTS being divided into two disease types: patients with a mutation in the* RECQL4* gene (RTS type 2) and patients without* RECQL4* gene mutation (RTS type 1). Approximately 66% of patients with RTS have a mutation in the* RECQL4* gene, which encodes one of the five proteins in the RecQ helicase family of proteins [5]. RecQ helicase proteins unwind DNA during replication or repair, and mutations in the* RECQL4* gene are believed to cause inaccurate DNA replication and repair leading to an increase in genome instability [5]. It is uncertain how this inaccurate DNA replication and repair lead to the specific physiologic abnormalities associated with RTS. While a specific gene mutation has not been identified for most of the other third of patients with RTS, it is believed that most of these patients likely have a mutation in another gene encoding a protein in the RecQ helicase family. Recently, in 2010, a second gene (*C16orf57*) mutation was identified in three families with RTS [6]. The* C16orf57* gene has been found to contribute to synthesis of U6 snRNA, which is important for gene splicing [7]. However, how mutations in the* C16orf57* gene specifically lead to the development of RTS and the prevalence of the* C16orf57* gene mutation in the RTS population remain unknown.

RTS is typically diagnosed in the first two years of life when the characteristic skin rash appears. No race or gender predilection for the disease has been identified. The incidence of RTS and carrier frequency of the* RECQL4* gene mutation is unknown; however, there have been less than 400 reported cases in the literature [8].

### 2.3. Clinical Findings

The diagnosis of RTS is made on clinical findings. The diagnosis of RTS can be made definitively if the characteristic poikilodermatous rash demonstrates the classic age of onset (between three and six months old), spread (beginning in the face and spreading to extensor surfaces of the extremities), and appearance (erythema/swelling gradually transitioning to chronic hyper- and hypopigmentation, telangiectasias, and skin atrophy) [8]. A diagnosis of probable RTS can be made if an atypical poikilodermatous rash is present in conjunction with two of the following findings: sparse scalp hair, sparse eyelashes and/or eyebrows, small stature, gastrointestinal disturbances in childhood, radial ray defects, radiographic bone abnormalities, dental anomalies, nail abnormalities, hyperkeratosis, juvenile cataracts, and osteosarcoma/skin cancers [8].

The characteristic poikilodermatous rash is invariably present, typically presenting before one year of age. Areas of decreased scalp hair or eyebrows/eyelashes are also common. Gastrointestinal symptoms such as chronic diarrhea or emesis have been reported in approximately 17% of patients. Although juvenile cataracts are one of the first described findings of RTS, it is a relatively uncommon finding being seen in less than 10% of patients [9].

### 2.4. Imaging Findings

While most of the findings of RTS will be elicited on clinical exam and history, there are several characteristic findings that will be encountered on imaging of the teeth and skeleton. Several types of dental abnormalities have been described in association with RTS, including rudimentary or hypoplastic teeth, microdontia, delayed eruption, supernumerary or absent teeth, ectopic eruption, and increased incidence of caries [10, 11]. Skeletal anomalies are seen in approximately 75% of patients, with anomalies including osteopenia, pathologic fractures, dislocations, patella ossification anomalies, metaphyseal irregularities, and radial limb abnormalities [9, 12]. A strong association has been found between mutations in the* RECQL4* gene and the presence of these skeletal anomalies [12].

Patients with RTS carry increased risk of developing both skin cancers and osteosarcoma. Osteosarcoma is the most common malignancy occurring in RTS, with an incidence as high as 30% reported in some studies [9]. A strong association has also been found between osteosarcoma and presence of the* RECQL4* gene mutation [13]. The appearance of osteosarcoma in RTS is similar to sporadic osteosarcoma with RTS patients having a slightly earlier mean age at diagnosis of between 9 and 12 years of age [13, 14].

### 2.5. Treatment and Prognosis

There is little published data on the life span of patients with RTS. However, in the absence of malignancy, the life expectancy is likely close to normal. Treatment of RTS primarily consists of preventative and surveillance care. Baseline skeletal radiographs by 5 years of age are recommended to identify underlying skeletal dysplasias, as are annual ophthalmic exams to identify cataracts. Calcium and vitamin D supplements may also be given in RTS patients that have bone demineralization or history of fractures. Patients are advised to avoid excessive sunlight, and close monitoring for changes in skin lesions is recommended due to the increased incidence of skin cancer. No screening recommendations for osteosarcoma have been described, but a low threshold for imaging is recommended in patients presenting with bone pain due to the increased risk for malignancy. The treatment recommendations for skin cancer and osteosarcoma are the same for RTS patients as for the rest of the population [8].

### 2.6. Differential Diagnosis

Two other syndromes are also associated with mutations of the* RECQL4* gene, RAPADILINO, and Baller-Gerold syndrome. Patients with RAPADILINO will often have osteopenia, small stature, radial ray defects, patellar hypoplasia, and GI abnormalities similar to RTS patients. However, RAPADILINO lacks the characteristic poikiloderma rash, with patients instead presenting with café au lait spots [8]. Baller-Gerold syndrome (BGS) also presents with osteopenia, short stature, radial ray defects, and other skeletal dysplasias similar to RTS, and patients with BGS may have poikiloderma. However, BGS patients frequently exhibit craniosynostosis, which is not typically associated with RTS [8, 15].

Osteogenesis imperfecta (OI) describes a group of heredity disorders of connective tissues characterized by increased bone fragility and decreased bone mineral density. OI is the most common cause of pediatric osteoporosis with a prevalence of approximately 1 in 12,000 to 15,000 children [16]. While some forms of OI present with multiple deforming fractures or perinatal death, the more retarded forms of the disease can present with short stature, osteoporosis, and only a few bone fractures, similar to RTS. However, OI is not associated with radial ray deformities; and wormian bones are present in the skull in approximately 60% of patients with OI [16], a feature not common in RTS. No specific skin changes have been described with OI. However, some forms of OI are associated with a blue sclera, which is a characteristic not seen in RTS.

## 3. Conclusion

Rothmund-Thomson syndrome is a rare autosomal recessive disorder most commonly characterized on imaging by dental and skeletal abnormalities, particularly radial ray defects. Skeletal abnormalities are particularly common in RTS patients with mutations in the* RECQL4* gene, and RTS patients with skeletal abnormalities are at increased risk for pathologic fragility fractures and delayed fracture healing due to associated diminished bone mineral density. Unfortunately RTS patients with the* RECQL4* gene mutation are also at a high risk of developing osteosarcoma, which may also present as a pathologic fracture. Therefore, careful consideration for underlying malignancy should be made in all fractures occurring in RTS patients.

## Abbreviations

BGS: Baller-Gerold syndrome
cm: Centimeters
FOV: Field of view
kVp: Peak kilovoltage
mAs: Milliampere-second
mL: Milliliters
mm: Millimeters
MRI: Magnetic resonance imaging
OI: Osteogenesis imperfecta
PD: Proton density
RTS: Rothmund-Thompson syndrome
ST: Slice thickness
TR: Relaxation time
TE: Echo time.
